# Effects of Subchronic Copper Poisoning on Cecal Histology and Its Microflora in Chickens

**DOI:** 10.3389/fmicb.2021.739577

**Published:** 2021-09-08

**Authors:** Cheng Huang, Yan Shi, Changming Zhou, Lianying Guo, Guohui Liu, Yu Zhuang, Guyue Li, Guoliang Hu, Ping Liu, Xiaoquan Guo

**Affiliations:** ^1^Jiangxi Provincial Key Laboratory for Animal Health, College of Animal Science and Technology, Jiangxi Agricultural University, Nanchang, China; ^2^School of Computer and Information Engineering, Jiangxi Agricultural University, Nanchang, China; ^3^Animal Husbandry and Veterinary Department of Ganzhou, Ganzhou, China

**Keywords:** Heavy metal pollution, copper, chicken, gut microbiota, 16S rDNA, metabolic disorders

## Abstract

Copper (Cu) is an important trace element with a two-sided effect on the growth performance of animals, which depends on the timing and dosage of Cu addition, etc. The purpose of this study was to determine the effects of oral copper sulfate (CuSO_4_, 350 ppm) on growth performance, cecal morphology, and its microflora of chickens (*n* = 60) after 30, 60, and 90 days. The results showed that after 90 days of copper exposure, the chickens lost weight, the cecum mucosa was detached, and vacuolation and inflammatory infiltration occurred at the base of the lamina propria. In addition, using the 16S rDNA sequencing method, we observed that copper exposure changed the richness and diversity of intestinal microorganisms. At the phylum level, *Proteobacteria* and *Actinobacteria* both significantly increased, while *Bacteroidetes* significantly decreased in the Cu group compared with control check (CK) group. At the genus level, the relative abundance of *Rikenellaceae_RC9_gut_group* decreased significantly, while *Ruminococcaceae_UCG-014, Lachnoclostridium*, and *[Eubacterium]_coprostanoligenes_group* increased significantly after copper exposure, and the change in microflora was most significant at 90 days. Moreover, the relevance of genus-level bacteria was altered. PICRUST analysis revealed potential metabolic changes associated with copper exposure, such as *Staphylococcus aureus* infection and metabolic disorders of nutrients. To sum up, these data show that subchronic copper exposure not only affects the growth and development of chickens but also causes the imbalance of intestinal microflora, which may further induce metabolic disorders in chickens.

## Introduction

Copper (Cu) is an essential trace element, which plays a key role in maintaining the normal growth and development of animals (Frieden, 1986). Cu can play a catalytic role as an important cofactor of various biological enzymes involved in immune function, protein synthesis, antioxidant defense, and energy metabolism. So adding high copper to diets has become a common practice to improve the production performance of livestock and poultry (Son et al., 2016; Berwanger et al., 2018). However, it has also been shown that Cu has the ability to inhibit immune response and increase the susceptibility of the host to pathogens (Zhao et al., 2018; Ruan et al., 2019). More importantly, early studies have shown that excessive copper intake may lead to the accumulation of copper in tissues, leading to organ and cytotoxic damage in animals and even humans, as well as intestinal damage (Pal, 2014). However, in order to improve production efficiency, copper is still being abused because it is often used as a promoter to inhibit fungal growth, bacterial and parasitic infections. Hence, people mostly ignore the toxic effect of excessive copper on the body. Additionally, with the development of industry, surface water and soil are also polluted by copper, which increases the risk of subchronic copper poisoning (Shahzad et al., 2012; Bui et al., 2016).

At present, more and more attention has been paid to the research on the effect of excessive copper intake on the body, and previous studies are mainly related to the following aspects. For instance, it was found that the addition of 796 mg/kg Cu to the diet of laying hens for 2 weeks would reduce the food intake, body weight, and egg yield of laying hens (Jackson et al., 1979). A previous study has also found that adding 50 mg/0.1 ml of copper sulfate to chicken embryos by injection can cause liver tissue necrosis and oxidative damage (Oguz et al., 2010). Copper exposure caused pathological damage to the liver and kidney of chickens, as well as changes in organ weight (Shahzad et al., 2012). Recently, some previous studies have reported that excessive copper intake increased the level of autophagy and apoptosis in chicken (Zhao et al., 2015, 2018; Fei et al., 2019). Another study suggested that subchronic copper exposure induced oxidative stress, resulting in mitochondrial disorder and jejunal toxicity increase due to redistribution of trace elements in the jejunum of chicken (Zhao et al., 2018). However, although there is a growing evidence reporting toxicity of heavy metal in chickens, very limited studies have been conducted to directly link toxicity to the changes in the gut microbiota.

The gastrointestinal tract (GIT) of chickens, especially the cecum, has a complex microflora. Intestinal microorganisms are closely related to the metabolism of the host, play a fundamental role in the health of the host, and benefit the host from many aspects, especially nutrition and disease resistance (Jenkins et al., 2019; Macchione et al., 2019). Although the composition of gut microbiota is relatively stable as the animal grows, it could be influenced by various factors, such as food, antibiotics, drugs, and even different environmental chemicals (Patterson and Burkholder, 2003; Gong et al., 2008). The evidence, to date, shows that a disruption of the normal intestinal microbiota in the host has been linked to disruption of physiological, metabolic homeostasis and promoting the development of various diseases to some extent, such as allergies, necrotizing enteritis, diabetes, depression, and cancer (Zhou C. et al., 2019). At present, studies have focused on the effects of copper exposure on intestinal microorganisms in mice (Ruan et al., 2019; Cheng et al., 2020). However, a few studies have explored the interactions between copper and microbiota in chicken, especially in the face of continuous excessive copper uptake. Therefore, it is crucial to research and understand the dynamic changes of intestinal microbial community in chicken during subchronic copper exposure.

Microbial 16S rDNA sequencing technology is an important means to study intestinal flora at the present stage (Jovel et al., 2016). Based on the technical progress of next-generation sequencing (NGS), it provides unparalleled coverage and depth in determining the intestinal dynamics of microorganisms (Mohd Shaufi et al., 2015). In the present study, we used this technique in assessing changes in intestinal bacterial communities induced by Cu exposure in chicken. The body weight and cecal morphology of chickens during subchronic copper poisoning were also observed and compared. We believed that the results obtained here can provide some new information for the toxicity of chicken caused by subchronic copper poisoning. Taken together, the objective of this study was to explore the toxic effects of subchronic copper poisoning on the intestinal tract of chickens.

## Materials and Methods

### Experimental Design and Animal Model

All the experimental schemes and methods used in this study were approved by the Animal Protection Agency and the use Committee (Jiangxi Agricultural University, Nanchang, Jiangxi, China), license No. JXAULL-2016038. The animals involved in the study were treated humanely, and steps were taken to minimize their suffering. One hundred and twenty Hy-line male chickens 1-day old (purchased from China Nanchang Guohua Co., Ltd.) were randomly divided into two groups (60 chickens in each group) after 1 week of breeding, including control group (CK) and the CuSO_4_-treated group (Cu). According to the test standard of subchronic poisoning in the Organization for Economic Co-operation and Development (OECD) guidelines and referring to the previous studies on the dose of copper poisoning in chickens, we set up the control group to drink tap water without CuSO_4_, the Cu group only drank CuSO_4_ working solution containing 350 ppm, and the whole experiment lasted 90 days (Council, 1995; Buschmann, 2013; Zhao et al., 2018). All chickens were free to drink feed and water. They received 12 h of light and 12 h of darkness every day. In addition, chickens were immunized according to routine immunization procedures.

On the 30th, 60th, and 90th day of the experiment, six chickens in each group were randomly weighed and euthanized with pentobarbital sodium. The cecal contents were quickly placed in liquid nitrogen and then stored at −80°C for experiment. At the same time, the cecal tissues were preserved in formalin for histopathological observation.

### Histopathological Analysis

The cecal tissues (∼0.3 cm^3^) were fixed in 4% paraformaldehyde, dehydrated in graded ethanol (70, 80, 90, and 100%) and xylene, and was embedded in paraffin. The tissue was circumcised to 5-μm thickness and stained with hematoxylin and eosin. Stained sections of feces were examined with a microscope (Olympus DP73, Japan) for morphological and structural changes in the mucosa.

### DNA Extraction and Quantitative Polymerase Chain Reaction Amplification

Total microbial genomic DNA from chicken feces was extracted using a combined cetyltrimethylammonium bromide/sodium dodecyl sulfate (CTAB/SDS) method including bacterial and fungal genomic DNA. Briefly, community DNA was extracted from a 0.25-g aliquot of each fecal sample. A 0.25-g aliquot of each fecal sample was extracted for community DNA. This DNA extraction method was employed: Two rounds of beating were performed in the presence of sodium chloride and sodium dodecyl sulfate, followed by sequential ammonium acetate and isopropanol precipitation. The precipitated nucleic acids were then treated with RNase A and Proteinase K, and the DNA was purified using columns of the QIAgen DNA Mini Stool Kit (QIAGEN, MD, United States). The DNA was then tested for purity and concentration by 1% agarose gel electrophoresis. An appropriate amount of DNA was taken in a centrifuge tube and diluted with sterile water to 1 ng/μl. Extracted metagenomic DNA was stored at −20°C.

The hypervariable region V3–V4 of 16S rDNA gene about 470 bp in length was sequenced. Polymerase chain reaction (PCR) was performed using specific primers 338F (5-GCACCTACTCCTACGGGAGCAGCAGCAGCA-3′) and 806R (5-GGACTACHVGGGGTWTCTAAT-3′). Polymerase chain reaction was performed with Q5 high-fidelity DNA polymerase at a final volume of 25 μl, and the template DNA was 20 ng. The thermal cycle included 98°C, 5 min (initial denaturation), 98°C, 10 s (denaturation), 50°C, 30 s (annealing), 72°C, 30 s (25 cycles), and 72°C, 5 min (final prolongation). The samples with the size of about 470 bp were selected by 2% agarose gel electrophoresis in 1.0 × TAE buffer, and then the amplified products were purified by AxyPrep DNA gel extraction kit (Axygen, AP-GX-250). The pyrosequencing of 16S rDNA was carried out on the Illumina HiSeq 2500 PE 250 platform (Illumina, San Diego, CA, United States) of Novogene Bioinformation Technology Co., Ltd. (Beijing, China).

### Sequence Processing and Bioinformatics Analysis

We used FLASH software (v1.2.7) to merge into a pair of end read and splice sequences to generate the raw tags. Open-source software system QIIME analysis was used to obtain high-quality clean tags, and UCHIME algorithm was used to remove the chimera sequence to obtain effective tags. The sequences were analyzed by UCLUST software and clustered into operational taxonomic units (OTUs) with 97% similarity. Each OTU was annotated with the Greengenes database. The rarefaction curve and Venn diagram were created with R software (v2.15.3). Microbial alpha diversity was analyzed by QIIME software with Python script. Beta diversity was evaluated by principal component analysis (PCA) to show the difference of bacterial community structure, and R (v2.15.3) was used to separate the bacteria by ANOSIM. Correlation analysis of genus-level bacteria using SparCC network maps. Prediction of the functional potential of bacterial community was done by PICRUST analysis.

### Data Presentation

The results were shown as mean and SEM. Significant difference was declared when *p* < 0.05. The 16S rDNA gene amplicon sequencing results were submitted to the Sequence Read Archive of the NCBI (accession number: SRP199017).

## Results

### Body Weight and the Morphology of the Cecum in Chicken

**FIGURE 1:**
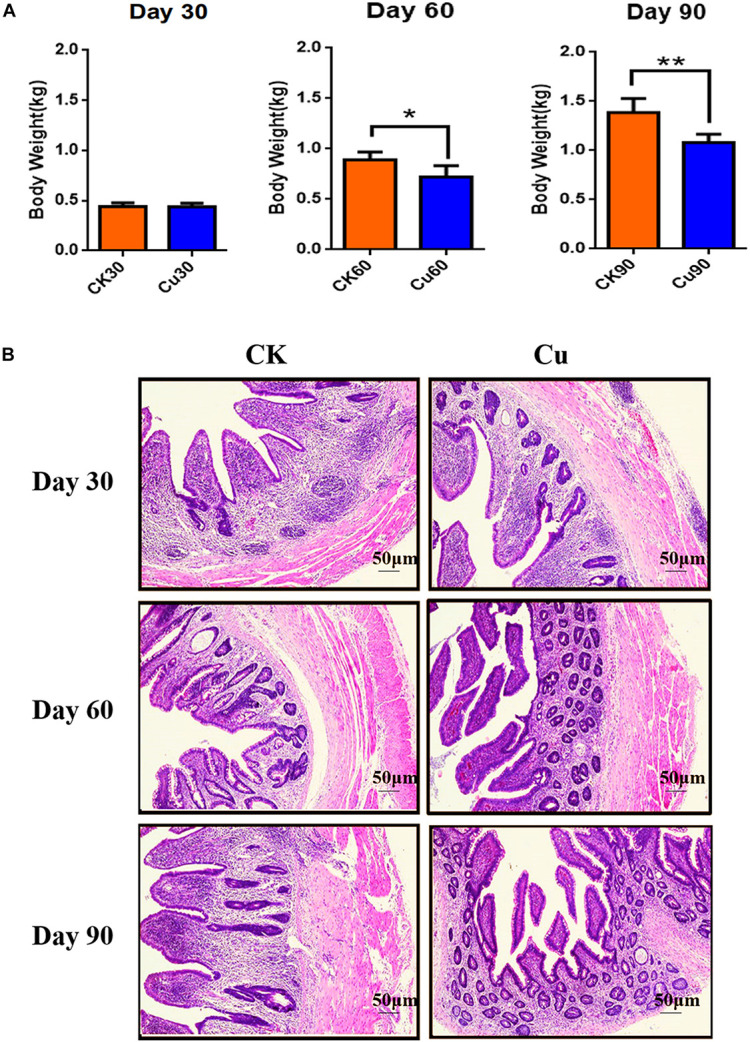
Defines weight and tissue samples at a specified time. Control check (CK): chickens in the control group were fed with copper-free distilled water. Oral copper sulfate (CuSO_4_)-treated group (Cu group): chickens drank CuSO_4_ water every day for 90 days. Body weight changes of chickens in **(A)** CK group and Cu group. **(B)** Representative images of hematoxylin and eosin (H&E) staining (original magnification, ×100) of chickens cecal tissue at different time points. All results are expressed as mean ± SD of six chickens in each group. * and ** represent statistically significant at *p* < 0.05 and *p* < 0.01 levels.

The weight of chickens were recorded at 30, 60, and 90 days, respectively, in order to evaluate the effect of CuSO_4_ intake on the growth performance of chickens. The results showed that compared with the CK group, the weight of the copper group decreased at all-time points, with significant difference at 60 days (*p* = 0.013) and 90 days (*p* = 0.000) (Figure 1A). The histological changes in the cecum in the CK group and Cu group at 30, 60, and 90 days are shown in Figure 1B. There were significant differences in the cecum tissue structure between the CK group and Cu group at different time points. In the whole process, no obvious lesion was found in the CK group, the cecal mucosa was intact, and the structure of the muscular layer was normal. Compared with the CK30 group, there was a small amount of mucosal exfoliation and vacuolation at the bottom of the lamina propria in the Cu30 group. Meanwhile, we found a large number of cecal mucosal exfoliation, lymphocyte proliferation, and vacuolation in the Cu60 group. On the 90th day, severe pathological changes occurred in the Cu group, with massive loss of cecal mucosa, inflammatory infiltration in the lamina propria, and destruction of the tissue structure of the muscular layer.

### Quality of Sequencing Analysis

**FIGURE 2:**
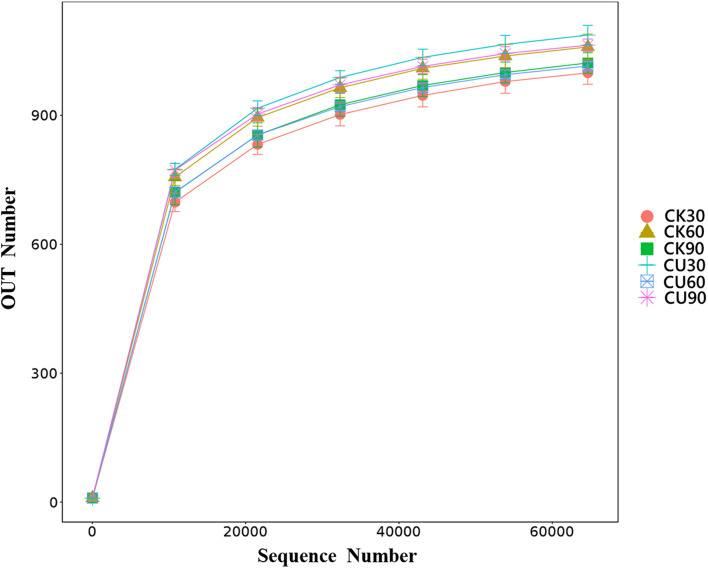
Rarefaction curve. The reads with similarity of 97% is clustered into the same service taxon operation taxonomic unit (OTU). The number of sequences sampled represents the number of sequencing reads.

Cecal samples (36) were sequenced by Illumina HiSeq platform and detected by 16S rDNA gene (V3–V4 region). A total of 2,871,406 sequences were obtained from these samples, of which 2,742,829 sequences were effective sequences by quality control filtration. After quality was checked and chimeric sequences were removed, the average number of reads generated from the cecal samples per chicken was 77,863 ± 5,525 [standard deviation (SD)], with an average length of 253 bp, respectively. They were classified using QIIME into different OTUs based on the identity level at 97%, and a total of 1,632 OTUs were identified from all samples. Shannon value and rarefaction curves for each sample reached the saturation plateau (Supplementary Table 1 and Figure 2), indicating that the samples had sufficient sequence coverage to accurately describe the bacterial composition of each group.

### Statistical Analysis of Alpha and Beta Diversity

**FIGURE 3:**
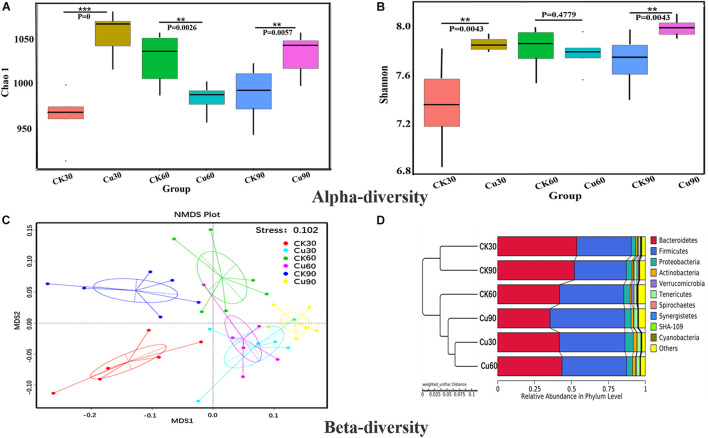
Alpha and beta diversity of cecal bacterial communities between control and Cu treated chickens. **(A)** Difference in bacterial diversity between CK and Cu groups was estimated by the Chao1 estimator at each time-point. The results were compared using the Wilcox test, *P < 0.05, **P < 0.01, and ***P < 0.001. **(B)** Difference in bacterial richness between CK and Cu groups was estimated by the Shannon value at each time-point. The results were compared using the Wilcox test, *P < 0.05, **P < 0.01, and ***P < 0.001. **(C)** Non-metric multidimensional calibration method (NMDS) generated using the weighted Uniface distance. **(D)** Cluster analysis performed on weighted Uniface distance matrices using the unweighted-pair group method with arithmetic mean (UPGMA).

Both alpha and beta diversity metrics were used to estimate microbial community diversity. The alpha diversity reflected the comprehensive index of richness and evenness of cecal flora in the CK and Cu groups. Beta diversity reflects whether there are significant differences in microbial communities between groups. In alpha diversity, the Chao1 index and ACE index represent community richness index, and community diversity index includes the Shannon index and Simpson index. Figure 3A shows the community richness index of the CK group and Cu group at three sampling times by the Chao1 index. The results showed that compared with the CK group, the community richness of the Cu group increased significantly after adding CuSO_4_ to drinking water for 30 days (Wilcox test, *p* < 0.001). After that, the community richness of the Cu group showed a downward trend, and at 60 days, compared with the CK group, the community richness of the Cu group decreased significantly (*p* < 0.01). In addition, compared with the CK group, the community richness of the Cu group increased significantly at 90 days (*p* < 0.01). Similarly, the Shannon index proved that the community diversity of the two groups had the same trend over time. This also confirms the results of previous studies, that is, heavy metal treatment can change the diversity of intestinal microbiota (Liu et al., 2014; Li et al., 2019). Changes in the intestinal microbial diversity may indicate that microbial diversity is disturbed, and intestinal health status is also changed (Wu et al., 2014).

Beta diversity uses the evolutionary relationship and abundance information between groups to calculate the distance matrix to reflect the differences between groups. Next, non-metric multidimensional calibration method (NMDS) based on weighted Unifrac distances was used to further determine the role of Cu intake in drinking water in changing the distribution of intestinal flora profile. As shown in Figure 3C, the NMDS ordination stress value is 0.102, and the microbial community of the Cu group was clearly separated from the CK group. In addition, the UPGMA method based on weighted Uniface is used to cluster the CK and Cu groups at different time points. Figure 3D shows that the individual samples of the CK group and the members of the Cu group gather in their respective groups and are significantly closer to each other at three time points. According to the results of the current beta diversity index, we speculated that the composition of microbial community also changed due to the change in copper exposure and time.

### Operational Taxonomic Unit Distribution From a Venn Plot

**FIGURE 4:**
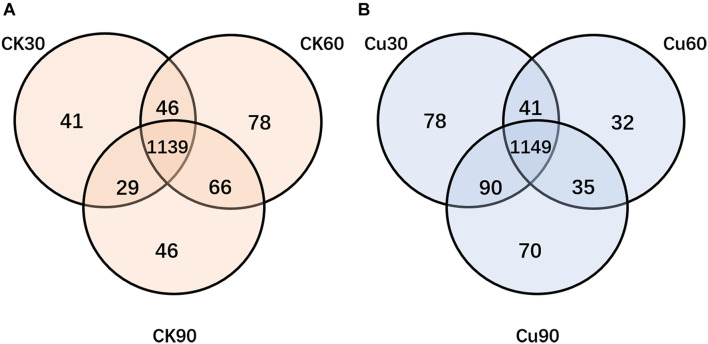
Venn diagram showing the number of unique and shared OTUs (at 97% similarity) among control and Cu-treated cecal microbial communities of chickens. **(A)** The number of unique and shared OTUs was observed in control group among three sampling times. **(B)** The number of unique and shared OTUs was observed in the Cu-treated group three sampling times.

In order to find out the difference between intestinal microbial community during the experiment, Venn diagrams were generated for the CK and Cu groups according to the results of OTUs cluster analysis. The Venn diagrams showed that, in the CK group, there were 1,139 OTUs common to all time point samples, and only 41, 78, and 46 OTUs were identified in the CK30, CK60, and CK90 samples, respectively (Figure 4A). In the Cu group, there were 1,149 OTUs that were common to all time points samples, and only 78, 32, and 70 OTUs were identified in the Cu30, Cu60, and Cu90 samples, respectively (Figure 4B). During the whole 90-day experimental period, the number of unique OTUS in the control group increased gradually in the first 60 days, but decreased during the last 30 days. In contrast, the number of unique OTUS in the exposed group decreased gradually in the first 60 days, but increased during the last 30 days. These results are consistent with the findings of the Chao1 index. Therefore, through these evidences, we have concluded that Cu exposure might change the cecal microflora of chickens.

### Composition Analysis of the Gut Microbiota at Various Taxonomic Levels

**FIGURE 5:**
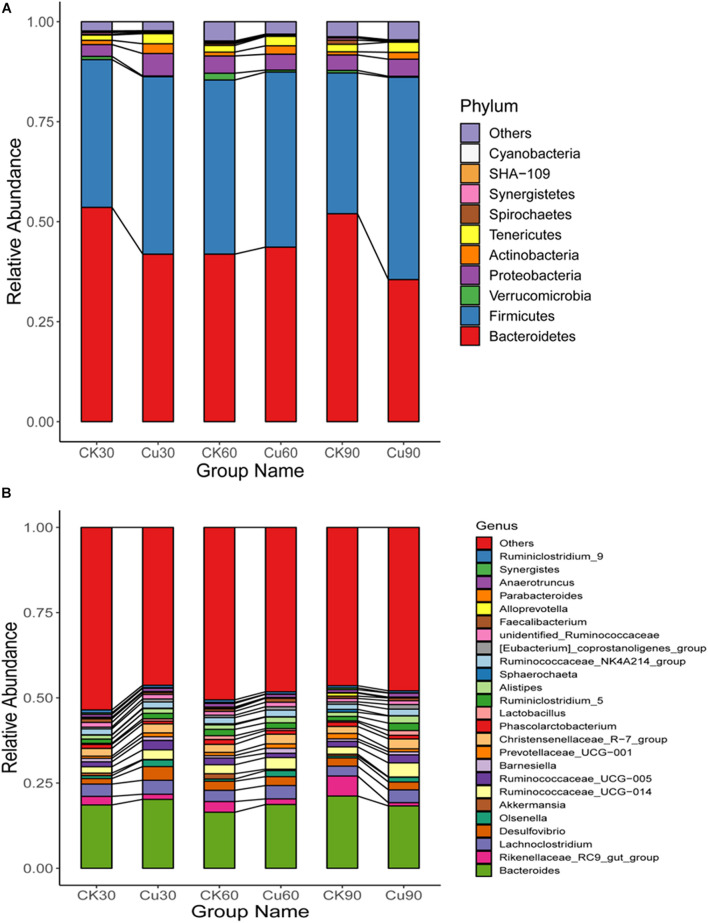
**(A)** Difference in the relative abundance of the bacterial major phyla between the CK and Cu groups during the experimental period (at days 30, 60, and 90). **(B)** Difference in the relative abundance of the major bacterial genera between the two groups during the experimental period (at days 30, 60, and 90).

According to the results of species annotation, each group of bacterial communities ranked at the level of phylum and genus were selected to form a column accumulation map of relative abundance of species, in order to observe the effect of copper exposure on cecal microorganisms during the experiment. At the phylum level, 10 taxa with high abundance of each sample were counted (Figure 5A) (mean relative abundance > 0.5%). *Bacteroidetes* and *Firmicutes* were found to be the most abundant phylum from each group in this study, with relative abundance of 44.75 and 42.40%, respectively (Figure 5A). While found in lower relative abundance, the phyla *Proteobacteria* (4.16%), *Tenericutes* (2.05%), and *Actinobacteria* (1.52%) were overall well represented across the experimental groups. Other minor phyla, such as *Verrucomicrobia* (0.67%), *Spirochaetes* (0.36%), *Synergistetes* (0.27%), *Cyanobacteria* (0.20%), and *SHA-109* (0.16%), were also identified. In addition, it was also found that the specific changes associated with CuSO_4_ exposure included a significant decrease in the relative abundance of *Bacteroides* in the Cu group on the 30th and 90th day compared with the CK group. Besides, phylum *Firmicutes* increased significantly on the 30th and 90th day, and had an increasing trend on the 60th day. Phylum *Proteobacteria* increased significantly on the 30th day, and *Tenericutes* increased significantly during the first 60-day period, and *Actinobacteria* increased significantly in the latter 60 days (Figure 5A and Supplementary Table 2). Consistent with the above results, compared with the middle 30-day period, the microflora of chickens exposed to CuSO_4_ changed more dramatically during the first 30-day period and the latter 30-day period.

At the genus level, 200 genera were identified from the two groups of chickens, and *Bacteroidetes* and *Rikenellaceae_RC9_gut_group* are the dominant genera of both the CK and Cu groups (Figure 5B). In addition, the relative abundance of *Ruminococcaceae_UCG-014*, *Desulfovibrio*, *[Eubacterium]_coprostanoligenes_group*, and *Lactobacillus* increased significantly after CuSO_4_ exposure for 30 days (Figure 5B and Supplementary Table 3). Compared with the CK group, the relative abundance of *[Eubacterium]_coprostanoligenes_group* and *Olsenella* increased significantly, and the relative abundance of the *Ruminococcaceae_UCG-005* decreased significantly after 60 days of CuSO_4_ treatment. It is noticeable that at 90 days, the cecal microorganisms in the Cu group changed dramatically, and the relative abundance of the genus from *Bacteroidetes*, *Firmicutes*, and *Actinobacteria* phylum, such as *Ruminococcaceae_UCG-014*, *Lachnoclostridium*, *Christensenellaceae_R-7_group*, increased significantly.

### Co-occurrence Network of Genera

**FIGURE 6:**
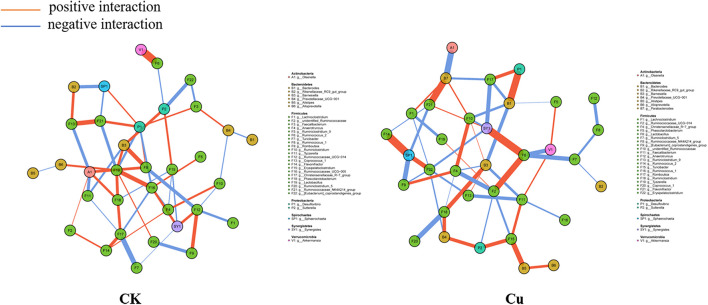
Co-occurrence network analysis of bacterial genera. Each network node represents a genus of bacteria. The connection width represents the relevant values that support this connection. The connection color shows correlation, red indicates positive interaction, and blue indicates negative interaction.

To explore potential interactions between members of the intestinal bacterial community, SparCC network maps were drawn based on genus composition and were considered valuable only if the association between the different genera was strong and the Spearman correlation coefficient was greater than 0.5 in absolute value and *q* < 0.05 (Figure 6). Overall, the co-occurrence network in the Cu group exhibited greater complexity with different components and correlations compared with the control group. Specifically, the co-occurrence network of the control group contains 106 edges; the co-occurrence network of the copper group contains 114 edges. We then calculated the closeness of the different bacteria at the genus level in the co-occurrence network. In terms of the tight centrality of nodes in the bacterial network, the three most correlated genera in both groups were *g__Lactobacillus*, *g__Ruminococcaceae_UCG-005*, *g__Desulfovibrio* in the normal group, *g__Synergistes*, *g__Sphaerochaeta*, and *g __Lactobacillus* in the copper group.

### Predictive Functional Profiling of Microbial Communities

**FIGURE 7:**
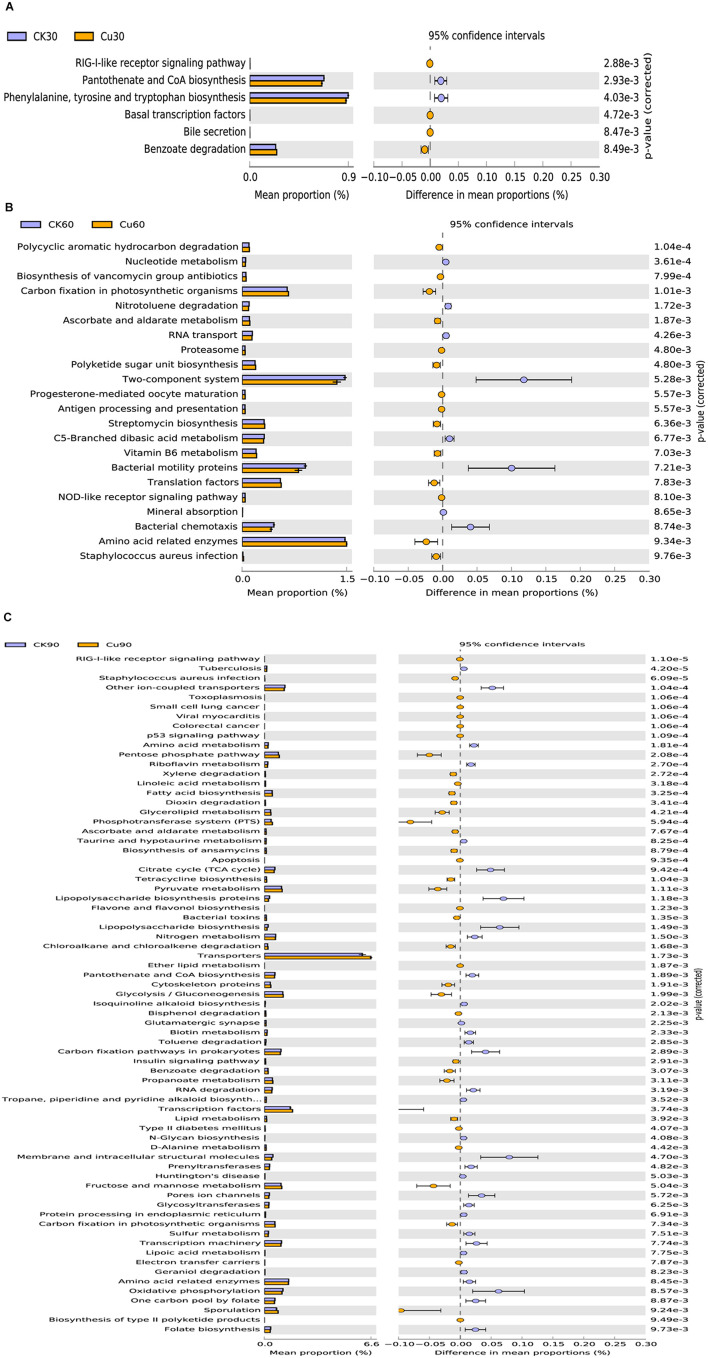
Functional analysis of microbial communities. KEGG categories predicted by PICRUSt (level 3) differentially represented between CK and Cu groups at day 30 **(A)**, day 60 **(B)**, and day 90 **(C)** during the experimental period. The statistical differences between the two groups at the same time point were conducted using a Welch’s *t*-test between the two groups; *p*-value < 0.01 was considered as statistical significant difference.

To further explore the functional changes in cecal microflora after CuSO_4_ exposure, the function of cecal microbial community was predicted and analyzed by PICRUSt. According to the annotated KEGG results, significant differences were observed between the control group and the CuSO_4_-treated group in the functional category of level 3, in which a strong separation was noted (Figure 7). On the 30th day after CuSO_4_ exposure, there were only six KEGG pathways, which showed significant changes between the CK30 and Cu30 groups. Figure 7A shows that compared with the CK30 group, the Cu30 group predicted higher gene abundance in the RIG-I-like receptor signaling pathway, basal transcription factors, bile secretion, and benzoate degradation, but lower predicted genes associated with pantothenate and CoA biosynthesis, and Phenylalanine, tyrosine, and tryptophan biosynthesis. At day 60, there were significant differences in more KEGG pathways between the two groups, a total of 22. The abundance of eight pathways such as nucleotide metabolism, RNA transport, bacterial motility proteins, and so on, in the CK60 group were upregulated, and the abundance of 14 pathways, such as polycyclic aromatic hydrocarbon degradation, biosynthesis of vancomycin group antibiotics, and ascorbate and aldarate metabolism were downregulated compared with those in the Cu60 group (Figure 7B). Finally, 90 days after CuSO_4_ exposure, the largest number of genomes met the inclusion criteria. A total of 71 KEGG pathways showed significant changes between the CK90 and Cu90 groups. Compared with the CK90 group, 36 pathways were upregulated, and 35 pathways were downregulated in the Cu90 group (Figure 7C). These pathway changes may be related to the prolonged exposure time to Cu.

## Discussion

Earlier, related studies have shown that high copper may cause damage to the liver, kidney, glandular stomach, and reproductive system of chickens, but there is still a lack of studies on the effects of copper exposure on intestinal damage and intestinal microorganisms in chickens (Jackson et al., 1979; Oguz et al., 2010; Zhao et al., 2018). In this study, we focused on the change in microflora in the cecum of chicken under the subchronic Cu exposure, and further revealed how the structure and composition of intestinal microorganisms evolved. Our results showed that subchronic copper poisoning in chickens caused weight loss, intestinal morphological change, and intestinal flora balance disorder.

The findings of this study suggest that compared with the control groups, the body weight of CuSO_4_-exposed chickens were decreased in a time-dependent manner. Previous studies have found that copper use of more than 300 mg/kg will lead to growth inhibition, showing an adverse effect on body weight (Pesti and Bakalli, 1996; Shahzad et al., 2012; Hamdi et al., 2018). A large number of histological injuries were also found in our histological observations. The results showed that copper exposure destroyed the normal structure of chicken cecum, mucosa fell off, vacuoles appeared in the lamina propria, and inflammatory infiltration occurred in a time-dependent manner. Interestingly, Ruan et al. (2019) and Cheng et al. (2020) demonstrated that copper exposure could cause intestinal mucosal damage and metabolic disorders, which reduce the decomposition and absorption of nutrients, and eventually lead to weight loss. Therefore, the pathological damage of cecum may be one of the reasons for the weight loss of copper-exposed chickens.

The chicken intestinal tract is inhabited by a complex and dynamic population of microbial species. Once there is long-term excessive intake of heavy metals, it will not only lead to morphological damage but also cause the disorder of the structure and composition of intestinal flora, and even lead to host metabolic diseases (Kou et al., 2019). According to the α diversity analysis of 16S rDNA sequencing results, copper exposure increased the richness and diversity of cecal microflora at 30 and 90 days and decreased at 60 days (Figures 3A,B), and, this is supported by Venn diagram analysis (Figure 4). These results showed that copper exposure causes significant changes in microbial diversity in the chicken cecum. The previous study suggests that it may be due to the fact that some bacteria can tolerate higher levels of copper and grow in copper-contaminated environments. Meanwhile, it was found by NMDS that the change in intestinal microflora was obvious between the two groups, and it depended on the time of exposure to CuSO_4_ (Figure 3C). The UPGMA analysis showed that the OTUs of the CK group was clustered into one group according to phylogeny, while that of the Cu group was clustered into another group (Figure 3D). The above results suggest that copper sulfate can disturb the diversity and composition of cecal flora in a time-dependent manner, which is consistent with previous studies suggesting that heavy metals can change intestinal flora (Cheng et al., 2020; Zhou et al., 2020). In addition, it has been reported that disorders of intestinal microflora is related to a variety of diseases such as intestinal barrier permeability and inflammation (Karlsson et al., 2013; Thevaranjan et al., 2017; Zhang et al., 2018). Therefore, we speculate that copper exposure may cause intestinal flora imbalance, which may have adverse effects on the health of chickens.

Previous studies suggest that *Bacteroidetes* and *Firmicutes* are considered to be the most and second common phyla in the intestinal microflora of birds, which is similar to our results (Figure 5A and Supplementary Table 2; Wei et al., 2013; Zhou et al., 2020). The results also showed that compared with the CK group, the abundance of *Proteobacteria* was increased in the Cu group after CuSO_4_ exposure. Several studies have reported that the abundance of *Proteobacteria* increased significantly when the intestinal mucosa was damaged (Michail et al., 2012; Tyler et al., 2013). In addition, we also noticed that copper exposure significantly increased the amount of *Tenericutes*. It has been reported that the amount of *Tenericuts* in colonic contents increased after 60 days of fluoride treatment, which may be related to the impairment of intestinal barrier function (Fu et al., 2019). Therefore, the above results showed that after Cu exposure, the balance of intestinal microorganisms at the phylum level is disrupted and may have high correlation with the intestinal infection in chicken.

Moreover, this study also reveals the potential effects of copper exposure on the structure of intestinal bacteria at the genus level. According to the results of species annotation, we found that the dominant bacteria in chicken cecum were *Bacteroides*, *Rikenellaceae_RC9_gut_group*, *Lachnoclostridium*, *Desulfovibrio*, and *olsenella* (Figure 5B). This was slightly different from the results of previous studies (Wei et al., 2013). One possible explanation for these results is that different intestinal environments may affect the abundance and composition of intestinal microflora. In addition, according to the *t*-test analysis, we noticed that *Desulfovibrio*, *Alistipes*, and *Anaerotruncus* increased significantly after copper exposure in 13 significantly changed genera, which is noteworthy (Supplementary Table 3). *Desulfovibrio* is a common genus in animals and humans. Its abundance increase is often accompanied by diarrhea, weight loss, and loss of appetite, which may further lead to intestinal epithelial hyperplasia, abscess, and inflammatory infiltration (Williamson et al., 2018). Moreover, Lau et al. (2006) demonstrated that *Anaerotruncus* may cause intestinal infection and bacteremia in the body. In addition, previous studies have found that *Alistipes* is considered to be a potentially harmful bacteria that may have an inflammatory effect (Kong et al., 2019). Based on the above observations, the results suggested that these genera with increased abundance in the copper group may play a partial role in intestinal injury and further cause intestinal barrier damage and intestinal inflammation. Therefore, this may explain why the body weight of the Cu group was lower than that of the CK group at all-time points.

The biological and physiological functions of intestinal microflora can be defined in many aspects, such as diversity and taxonomic composition, in order to decipher the potential role of intestinal bacterial community (Liu et al., 2019). In the current experiment, the PICRUST algorithm is used to map the bacterial genetic path to the KEGG database for functional prediction. A deeper analysis discovered that copper sulfate may change the microbiome and metabolic spectrum of chickens in a time-dependent manner. Compared with the CK group, a wide range of pathways were significantly changed under the influence of subchronic copper poisoning in the Cu group, including multiple gene functional families, such as energy production and conversion, amino acid transport and metabolism, signal transduction mechanism, etc. The results also showed that compared with the CK group, the relative abundance of genes regarding amino acid metabolism is decreased, and carbohydrates and lipids are increased in the Cu group (Figure 7). Interestingly, previous studies had confirmed that bacterial functional pathways in inflammatory tissues of patients with ulcerative colitis were significantly changed, carbohydrate metabolism decreased, and lipid and amino acid metabolism increased (David et al., 2014). Therefore, this may explain the reason for the difference, which may be related to the host and CuSO_4_ exposure models used in the study. Likewise, it was worth noting that compared with the control group, the cecal microbial community in the copper group showed increased relative abundance of genes regarding bacterial toxin, apoptosis, *Staphylococcus aureus* infection, sporulation, nucleotide metabolism, and so on, and downregulation of lipoic acid and lipopolysaccharide biosynthesis in sulfur metabolism. This is consistent with previous studies and verified the morphological changes in the cecum in chickens (Zhou L. et al., 2019).

## Concluding Remarks

In short, we found that subchronic copper exposure could lead to weight loss, pathological changes in cecal tissue, change the diversity, structure, and function of microbial community, and finally destroy the balance of intestinal microbial community in chickens. To sum up, our results may play a role in the effects of copper exposure on intestinal microorganisms in chickens.

Subchronic copper exposure disrupts the environmental homeostasis of intestinal microbes and alters the structure and balance of the microbial community (Jovel et al., 2016). Furthermore, alterations in intestinal flora may lead to changes in the intestinal environment such as acidity and alkalinity, intestinal infection, intestinal barrier permeability, and inflammation (Gong et al., 2008; Wei et al., 2013). Long-term effects can disrupt the normal structure of the chicken cecum, with loss of intestinal villi and metabolic disturbances, which may ultimately lead to weight loss (Shahzad et al., 2012; Ruan et al., 2019).

## Data Availability Statement

The datasets presented in this study can be found in online repositories. The names of the repository/repositories and accession number(s) can be found below: https://www.ncbi.nlm.nih.gov/, SRP199017.

## Ethics Statement

The animal study was reviewed and approved by the Jiangxi Agricultural University, Nanchang, Jiangxi, China, license No. JXAULL-2016038.

## Author Contributions

CH, YS, CZ, LG, GLiu, YZ, GLi, GH, PL, and XG contributed to the conception of the review. CH, YS, PL, and XG drafted the manuscript. All authors reviewed and approved the manuscript.

## Conflict of Interest

The authors declare that the research was conducted in the absence of any commercial or financial relationships that could be construed as a potential conflict of interest.

## Publisher’s Note

All claims expressed in this article are solely those of the authors and do not necessarily represent those of their affiliated organizations, or those of the publisher, the editors and the reviewers. Any product that may be evaluated in this article, or claim that may be made by its manufacturer, is not guaranteed or endorsed by the publisher.
