# Supplementary figures and images for: Effects of Subchronic Copper Poisoning on Cecal Histology and Its Microflora in Chickens
Source: Front Microbiol. 2021 Sep 8;12:739577. doi: 10.3389/fmicb.2021.739577 (PMC8456085; doi:10.3389/fmicb.2021.739577)

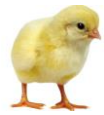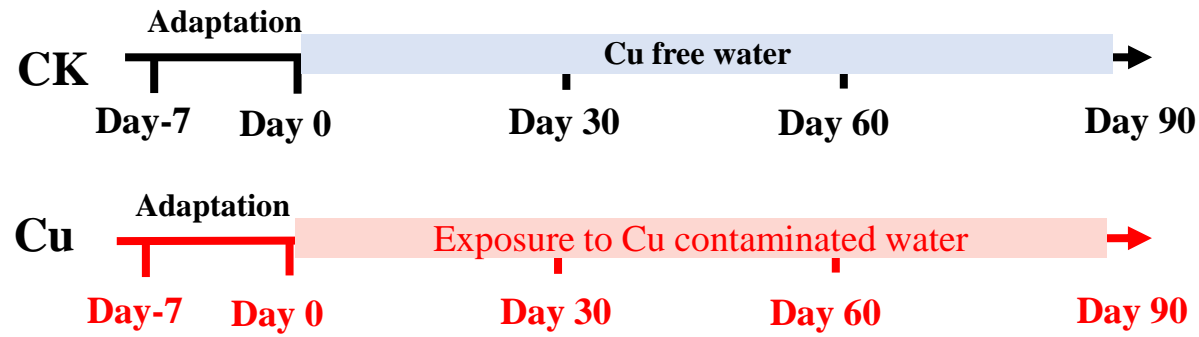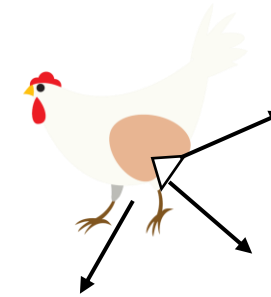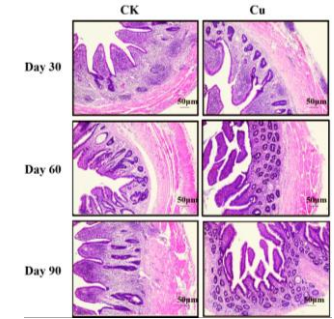

Cecal tissue structure injury

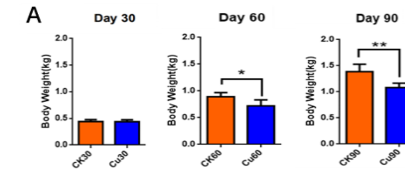

Body weight decline

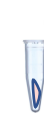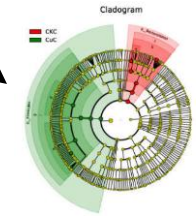

16S rDNA sequencing

Supplement: Supplementary file 1 [file Image_1.PDF]
