# Supplementary material for: Effects of Subchronic Copper Poisoning on Cecal Histology and Its Microflora in Chickens
Source: Front Microbiol. 2021 Sep 8;12:739577. doi: 10.3389/fmicb.2021.739577 (PMC8456085; doi:10.3389/fmicb.2021.739577)

Table S1. Data preprocessing statistics and quality control.


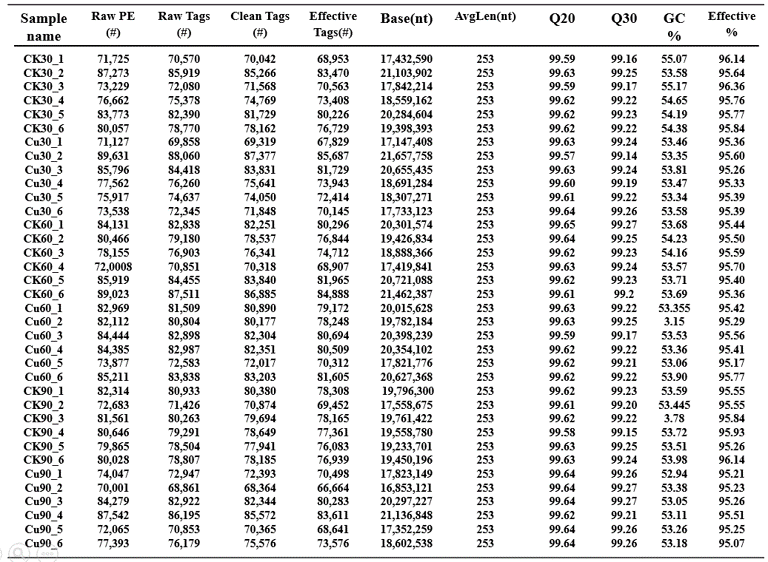


Table S2. Average relative abundance (%) of dominant phyla in cecal microbiota of chicken at 30 days, 60 days, and 90 days after exposure to CuSO_4_.


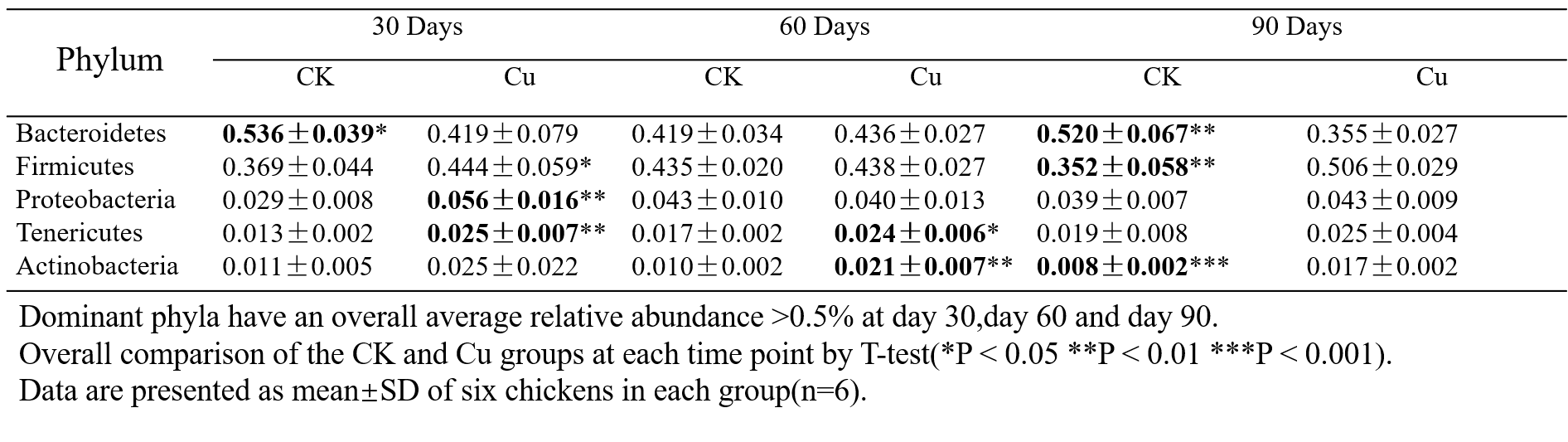


Table S3. Average relative abundance (%) of dominant genera in cecal microbiota of chicken at 30 days, 60 days, and 90 days after exposure to CuSO4.


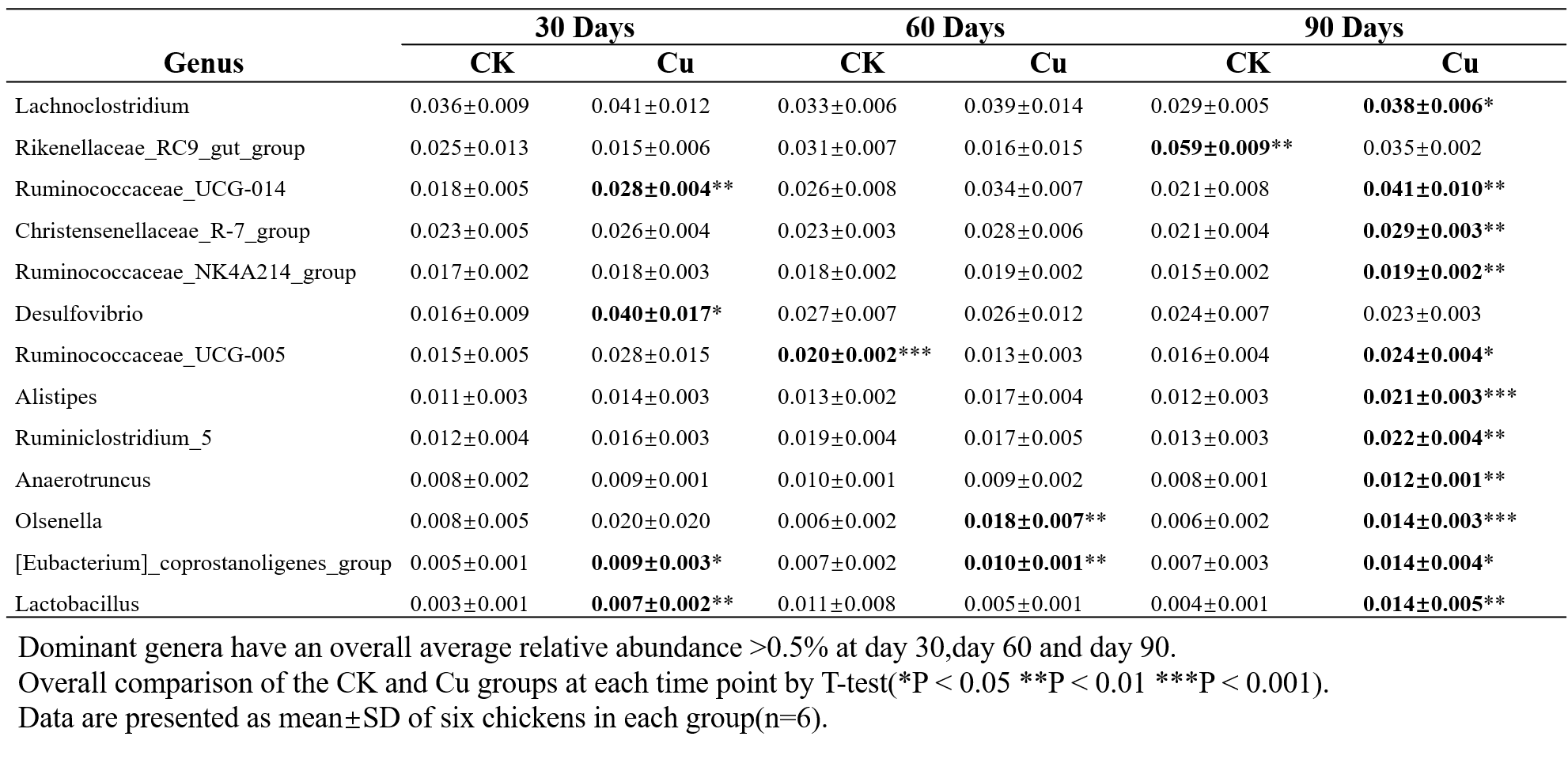

Supplement: Supplementary file 2 [file Table_1.docx]
